# Identification of Structural Features for the Inhibition of OAT3-Mediated Uptake of Enalaprilat by Selected Drugs and Flavonoids

**DOI:** 10.3389/fphar.2020.00802

**Published:** 2020-05-28

**Authors:** Yao Ni, Zelin Duan, Dandan Zhou, Shuai Liu, Huida Wan, Chunshan Gui, Hongjian Zhang

**Affiliations:** College of Pharmaceutical Sciences, Soochow University, Suzhou, China

**Keywords:** three-dimensional quantitative structure-activity relationship, enalaprilat, flavonoid, herb-drug interaction, organic anion transporter 3 (OAT3)

## Abstract

Enalaprilat is the active metabolite of enalapril, a widely used antihypertension drug. The human organic anion transporter 3 (OAT3), which is highly expressed in the kidney, plays a critical role in the renal clearance of many drugs. While urinary excretion is the primary elimination route of enalaprilat, direct involvement of OAT3 has not been reported so far. In the present study, OAT3-mediated uptake of enalaprilat was first characterized, and the inhibition of OAT3 transport activity was then examined for a number of flavonoid and drug molecules with diverse structures. A varying degree of inhibition potency was demonstrated for flavonoids, with IC_50_ values ranging from 0.03 to 22.6 µM against OAT3 transport activity. In addition, commonly used drugs such as urate transporter 1 (URAT1) inhibitors also displayed potent inhibition on OAT3-mediated enalaprilat uptake. Pharmacophore and three-dimensional quantitative structure-activity relationship (3D-QSAR) analyses revealed the presence of a polar center and a hydrophobic region involved in OAT3-inhibitor binding. For the polar center, hydroxyl groups present in flavonoids could act as either hydrogen bond donors or acceptors and the number and position of hydroxyl groups were critical drivers for inhibition potency, while carboxyl groups present in some drugs could form ionic bridges with OAT3. The predicted inhibition potencies by comparative molecular field analysis (CoMFA) and comparative molecular similarity indices analysis (CoMSIA) were correlated well with experimental IC_50_ values. Taken together, the present study identified OAT3-mediated uptake of enalaprilat as an important mechanism for its renal clearance, which may be liable for drug-drug and herb-drug interactions. The established computational models revealed unique structural features for OAT3 inhibitors and could be used for structure-activity relationship (SAR) analysis of OAT3 inhibition. The clinical relevance of the inhibition of OAT3-mediated enalaprilat uptake warrants further investigation, particularly in populations where herbal remedies and drugs are used concomitantly.

## Introduction

Natural remedies containing flavonoids and functional components are widely consumed to maintain health status and vigor. In aging populations where chronic ailments (hypertension, hyperlipidemia, hyperuricemia, etc.) are prevalent, prescribed medicines, over the counter agents, and natural products including functional food components are often used concomitantly. Consequently, herb-drug interaction represents an underlying risk as some natural components may alter pharmacokinetics, efficacy, and safety of therapeutic agents (Agbabiaka et al., 2018; Briguglio et al., 2018; Parvez and Rishi, 2019). As such, investigations on disposition mechanisms of xenobiotics and their association with herb-drug interaction can provide scientific foundations for safe and effective use of various remedies.

Enalapril, an orally active inhibitor of the angiotensin converting enzyme (ACE), is widely used for the treatment of hypertension and heart failure. Enalapril is a prodrug which is hydrolyzed by carboxylesterase 1 (CES1) to form the active metabolite, enalaprilat (Ulm et al., 1982). It has been shown that polymorphism of CES1 affects the pharmacokinetics and pharmacodynamics of enalapril in human subjects (Tarkiainen et al., 2015; Stage et al., 2017). Enalapril is rapidly absorbed with about 40% of the dose (10 mg) converted to enalaprilat (Ulm et al., 1982). Both enalapril and enalaprilat are excreted into the urine, suggesting the importance of renal clearance in the disposition of the drug and its active metabolite (Ulm et al., 1982; MacFadyen et al., 1993).

Renal clearance of drugs and endogenous substances is generally governed by three major processes: filtration, carrier-mediated excretion, and carrier-mediated reabsorption. In the realm of carrier-mediated excretion, organic anion transporters (OAT), particularly organic anion transporter 3 (OAT3), has been shown to play an important role in the renal clearance of many drugs and endogenous substances (Burckhardt, 2012; Nigam, 2018). Recent evidence indicates that OAT3-mediated renal clearance can be inhibited by numerous drugs and natural products (Ivanyuk et al., 2017; Wang et al., 2018), leading to unwanted alterations in pharmacokinetics, efficacy, and safety of underlying therapeutic agents.

Role of drug transporters in the disposition of enalapril and its active metabolite enalaprilat has not been fully appreciated. Early reports suggested that the liver uptake of enalapril could be mediated by organic anion transporting polypeptide (OATP) 1B1 (Abu-Zahra et al., 2000; Tian et al., 2011). With regards to carrier (transporter)-mediated renal clearance, *in vitro* evidence implied the presence of a “barrier” for the kidney entry of enalaprilat (de Lannoy et al., 1989), while the exact mechanism was unclear. A human pharmacokinetic study indicated that probenecid could markedly increase systemic exposure of enalapril and enalaprilat by decreasing their renal excretion (Noormohamed et al., 1990). As probenecid is a well characterized inhibitor of OAT3 (Tahara et al., 2006; Zhou et al., 2020), it could be assumed that the interaction between probenecid and enalapril/enalaprilat might be associated with OAT3-mediated renal clearance, although no direct evidence on OAT3’s involvement was described.

In the present study, we first identified OAT3 as an important uptake transporter for enalaprilat and characterized its transport kinetics in stably transfected cell lines. Inhibition of OAT3-mediated uptake of enalaprilat was then examined and characterized for a number of drugs and flavonoids. In addition, pharmacophore and three-dimensional quantitative structure-activity relationship (3D-QSAR) analyses were performed to identify structural features for OAT3-inhibitor binding. Results indicated that the computational models established could be useful tools for structure-activity relationship (SAR) analysis of OAT3 inhibition.

## Materials and Methods

### Materials

Enalapril maleate was purchased from Tokyo Chemical Industry Co., Ltd. (Shanghai, China). Enalaprilat, gemfibrozil, telmisartan, repaglinide, glimepiride, febuxostat, valsartan, and diclofenac sodium were purchased from Sigma-Aldrich (St. Louis, MO, USA). Dulbecco’s modified Eagle’s medium (DMEM) and fetal bovine serum (FBS) were purchased from Hyclone (Logan, UT, USA) and trypsin was from Genom (Hangzhou, China). Twenty four-well plates biocoated with poly-D-lysine was obtained from BD Biosciences (San Jose, CA, USA). Hanks’ balance salt solution (HBSS) containing 1.3 mM CaCl_2_, 0.5 mM MgCl_2_, 0.4 mM MgSO_4_, 5.4 mM KCl, 0.4 mM KH_2_PO_4_, 137 mM NaCl, 4.2 mM NaHCO_3_, 0.3 mM Na_2_HPO_4_, 10 mM HEPES, and 5 mM D-glucose was prepared in house. All other reagents and chemicals were of analytical grade or of the highest quality available commercially.

### Cell Culture

Human embryonic kidney 293 (HEK293) cells stably overexpressing human OAT1, OAT3, organic cation transporter 2 (OCT2), OATP1B1, OATP1B3 or OATP2B1 as well as empty vectors were generously provided by Professor Dafang Zhong, Shanghai Institute of Materia Medica, Chinese Academy of Sciences (Shanghai, China). Respective transport activities had been validated using specific probe substrates (Zhong et al., 2014). All cells were grown in DMEM supplemented with 10% FBS, at 37°C with 5% CO_2_ and 95% humidity.

### Transporter-Mediated Uptake of Enalaprilat

Cellular uptake of enalaprilat was evaluated using HEK293 cells overexpressing individual human uptake transporters. Cells were seeded in 24-well plates at a density of 200,000 cells per well and cultured in high glucose DMEM for 48 h. On the third day and prior to the start of the uptake experiment, cells were washed thrice with pre-warmed HBSS and then incubated with HBSS for 30 min at 37°C. Uptake experiments were carried out by the addition of 500 μl HBSS containing defined concentrations of the substrate enalaprilat (0, 1, 3, 10, 30, 100, 500 µM). Incubations were terminated 5 min after the addition of substrate by washing with ice-cold HBSS. Cells were immediately lysed with 200 μl ultrapure water by repeated freezing and thawing up to three times. Intracellular drug concentrations were determined using a liquid chromatography - tandem mass spectrometry (LC-MS/MS) method. Cellular protein levels were estimated using the Bicinchoninine acid assay per manufacturer’s instruction (Takara, Japan). Active uptake of enalaprilat by individual transporter was obtained by subtracting cellular concentrations in the control cells (Mock) from the transporter transfected cells to correct for possible contribution of passive diffusion and/or background transport activity.

### Inhibition of OAT3-Mediated Enalaprilat Uptake

Based on results from the above uptake studies, OAT3 was identified as an important uptake transporter for enalaprilat. Subsequently, the inhibition of enalaprilat uptake by selected flavonoids and drugs was examined using OAT3-transfected HEK293 cell lines. As reported in the literature, inhibitory potency of certain compounds toward OAT3 transport activity might be enhanced after a period of preincubation and the inhibitory potency increase was compound dependent (Ma et al., 2015). Because the present study examined a number of compounds with structural diversity, a 30 min preincubation was carried out for all compounds in order to eliminate inconsistencies. Inhibition assays were performed in 24-well poly-D-lysine-coated plates using enalaprilat as a substrate (30 μM, below the K_m_ value). OAT3-transfected HEK293 cells or mock cells were washed thrice with pre-warmed HBSS and preincubated with HBSS containing different concentrations of inhibitors for 30 min at 37°C. After 30 min, the preincubation medium was aspirated and cells were then incubated with HBSS containing the same concentrations of inhibitors and substrate enalaprilat to initiate the uptake inhibition study. Inhibition potency was first screened at a single concentration of inhibitors (100 μM) to triage compounds and IC_50_ values were then determined for selected flavonoids and drugs showing strong inhibition against OAT3-mediated uptake of enalaprilat. Mean and standard deviation of enalaprilat concentrations were calculated for each group of measurements (N=3). In all experiments, active uptake of enalaprilat was obtained by deducting cellular concentrations in the control cells (Mock) from the individual transporter transfected cells as described previously.

### Pharmacophore Modeling

Pharmacophore modeling calculations were carried out by using the GALAHAD module implemented in Sybyl-X 2.0 software (Tripos International, St. Louis, MO), which was operated in two main stages (Richmond et al., 2006). It first used a genetic algorithm (GA) to identify a set of ligand conformations that minimized energy while maximizing pharmacophore multiplet similarity between ligands. The ligands were flexibly and fully aligned to each other in internal coordinate space in this stage. Then a rigid-body alignment was performed for the produced conformations in Cartesian space (Shepphird and Clark, 2006). The features considered in developing the pharmacophore model included hydrogen bond donor (HBD) atoms, hydrogen bond acceptor (HBA) atoms, hydrophobic, and charged centers. In this study, five drugs containing acid moieties and nine flavonoids were selected to generate the pharmacophores for organic anions and flavonoids, respectively. The selected compounds were strong OAT3 inhibitors with IC_50_ values smaller than 1 μM. Compounds were aligned with each other using suggested values for GALAHAD parameters.

### 3D-QSAR Analysis of OAT3 Inhibitors

Conformational determination and structural alignment were performed for tested flavonoids as OAT3 inhibitors. Structures of flavonoids were sketched in Sybyl-X 2.0 software program and the initial conformations were generated by molecular mechanic optimization using the Tripos force field and Gasteiger-Hückel charges (Gasteiger and Marsili, 1980), with an energy gradient convergence criterion of 0.05 kcal/mol and a distance-dependent dielectric constant of 1. After that, conformational search with acyclic rotatable bonds was performed for each flavonoid by Confort program (Pearlman and Balducci, 1998). The conformation with lowest energy was selected and subjected to energy minimization until convergence. The resulting conformation was used for molecular alignment. Initial alignment was obtained by aligning the flavonoids according to their backbone structures. Conformational adjustment by rotating the rotatable bonds was performed for representative flavonoids with visual inspection. However, all rotations were confined within 10 kcal/mol of its lowest energy.

The inhibition potency toward OAT3 was expressed in pIC_50_ (−logIC_50_) values, which were used as the dependent variables in comparative molecular field analysis (CoMFA) and comparative molecular similarity indices analysis (CoMSIA) (Klebe and Abraham, 1999). In CoMFA analysis, steric and electrostatic field energies were probed with a sp^3^ carbon atom having a charge of +1. Steric and electrostatic interactions were calculated using a Tripos force field with a distance-dependent dielectric constant. A cutoff value of 30 kcal/mol was applied on steric and electrostatic fields. Column filtering was set to be of 1.0 kcal/mol. In CoMSIA analysis, five different similarity fields, namely steric, electrostatic, hydrophobic, hydrogen-bond donor, and hydrogen-bond acceptor fields, were calculated with a probe atom having a radius of 1 Å, charge of +1, hydrophobicity of +1, and hydrogen bonding donor and acceptor properties of +1. These fields covered the major contributions to ligand binding. The attenuation factor was set to be of 0.3. The cross validated r^2^ (q^2^) and optimum number of components were obtained by the partial least-squares (PLS) method with the leave-one-out option. With the obtained optimum numbers of components, the final non-cross validated CoMFA and CoMSIA models were developed.

### Quantitation of Drug Concentrations by LC-MS/MS

For sample preparation before analysis, 100 μl acetonitrile containing 500 nM tolbutamide (internal standard, IS) was added as a protein-precipitating agent to 50-μl cell samples. All samples were vortexed for 2 min and centrifuged at 13,000 rpm for 10 min. Ten microliters of the supernatants were transferred into injection vials for concentration determination. Enalaprilat was shown to be stable after three freeze-thaw cycles, short- and long-term storage and under post-preparative conditions (Ramusovic et al., 2012).

Concentrations of enalaprilat were determined by using LC-MS/MS. The LC-MS/MS system consisted of an API 4000 Qtrap mass spectrometer equipped with a turbo-V ionization source (Applied Biosystems, Foster City, CA, USA), two LC-20AD pumps with a CBM-20A controller, a DGU-20A solvent degasser, and a SIL-20A autosampler (Shimadzu, Columbia, MD, USA). A reverse phase C_18_ column (50×2.1 mm, 5 µm particle size; Agela, China) and a gradient elution at 0.3 ml/min were employed for chromatographic separation. Mobile phases were comprised of mobile phase A containing 95% water, 5% acetonitrile, and 0.1% formic acid and mobile phase B with 95% acetonitrile, 5% water, and 0.1% formic acid. A gradient elution program was carried out with mobile phases as follows: 0–1 min (95% A, 5% B), 1–1.5 min (95–20% A, 5–80% B), 1.5–3.0 min (20% A, 80% B), 3.0–3.1 min (20–95% A, 80–5% B) and 3.1–5 min (95% A, 5% B). Enalaprilat was analyzed with multiple reaction-monitoring (MRM) in the electrospray ionization (ESI) positive mode. The selected transitions, declustering potential (DP) and collision energy (CE) were as follows: m/z 349.2 → 206.1, DP 80 V and CE 26 eV for enalaprilat; m/z 271.1 → 172.1, DP 70 V and CE 18 eV for tolbutamide (IS), respectively. Analyst 1.6.3 (AB SCIEX, USA) was used to collect and process the data.

### Data Analysis

Kinetic parameters of transporter-mediated uptake were estimated by nonlinear regression analysis using GraphPad Prism 5.01 (La Jolla, CA, USA). Equations used were as follows:

$$\text{v}=\frac{{\text{V}}_{\text{max}}\times [\text{S}]}{{\text{K}}_{\text{m}}+[\text{S}]}$$

where v was the rate of uptake, V_max_ was the maximum rate, [S] was the substrate concentration. K_m_ was the Michaelis constant.

For inhibition studies, IC_50_ values were calculated from semi-logarithmic plots of the inhibitor concentrations vs. percentage of net uptake relative to the control using GraphPad Prism Version 5.01:

$$\%\text{Control}=100/(1+\text{I}/{\text{IC}}_{50})$$

where I was the inhibitor concentration.

Data were presented as mean ± sd and differences were considered significant when p<0.05. The unpaired two-tailed Student’s t-test was used for statistical analysis.

## Results

### Transporter-Mediated Uptake of Enalaprilat

**Figure 1:**
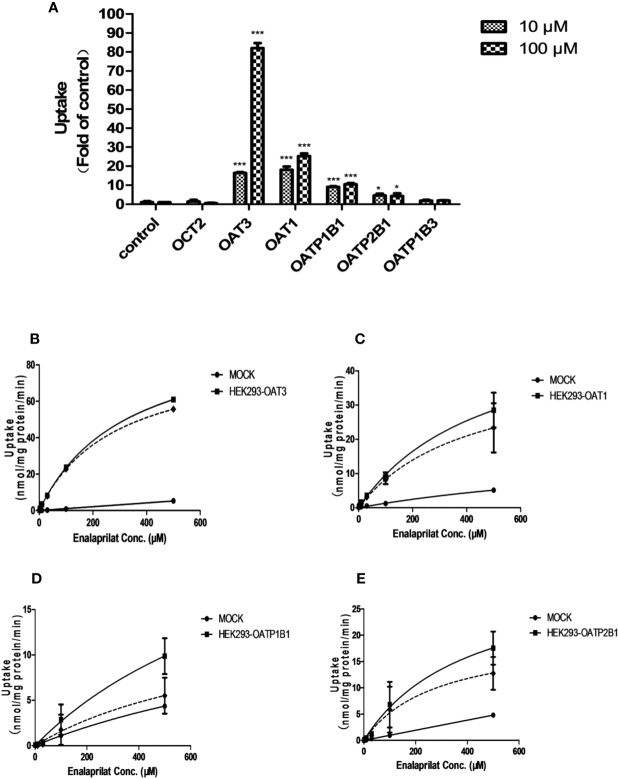
Uptake of enalaprilat in HEK293 cells transfected with individual uptake transporters. Enalaprilat uptake by human OCT2, OAT3, OAT1, OATP1B1, OATP2B1, and OATP1B3 at 10 and 100 µM **(A)**; Uptake transport kinetics of enalaprilat mediated by **(B)** OAT3, **(C)** OAT1, **(D)** OATP1B1, and **(E)** OATP2B1. The dashed lines represented “net transporter specific uptake” calculated by subtracting mock cell concentrations from the total at each time point (Section 3.3). Data were presented as mean ± sd. *P < 0.05, ***P < 0.001.

The uptake of enalaprilat was evaluated first using HEK293 cell lines stably transfected with human uptake transporters including OAT1, OAT3, OCT2, OAPT1B1, OATP2B1 and OATP1B3, at two concentrations (10 and 100 μM). As illustrated in **Figure 1A**, OAT3 was initially identified as a likely important transporter that mediated the uptake of enalaprilat. While passive diffusion and/or other transporter-mediated processes was noticed at a very low level (control group), the uptake of enalaprilat mediated by OAT3 was particularly evident at high concentration. In addition, OAT1, OATP1B1, and OATP2B1 were also involved in the enalaprilat uptake albeit to lesser extents. Similarly, enalapril could also be transported by OAT3 (**Supplementary Figure 1**). Because enalapril is a prodrug and its plasma exposure is about 4.5-fold lower than that of enalaprilat (Li et al., 2017), subsequent studies were focused on enalaprilat, the pharmacologically active compound after enalapril administration.

Uptake kinetics of enalaprilat was investigated in transfected cell lines mentioned above, and concentration-dependent uptake and kinetic parameters were shown in **Figures 1B–E** and summarized in **Table 1**. Judging from V_max_/K_m_ ratio (an indicator for the intrinsic uptake clearance), the role of human transporters in enalaprilat uptake followed the order of OAT3 >> OAT1 > OATP2B1 > OATP1B1. These kinetic results further confirmed the predominant roles of OAT3 in the carrier-mediated uptake and renal clearance of enalaprilat.

**Table 1:** Kinetic parameters of enalaprilat uptake mediated by human OAT3, OAT1, OATP1B1, and OATP2B1^a^.

Cell Lines	Kinetic Parameters^b^		
	V_max_ (nmol/min/mg protein)	K_m_ (μM)	CL_int_^c^ (ml/min/mg protein)
OAT3	87.4 ± 1.1	284.5 ± 8.2	0.307
OAT1	42.4 ± 1.3	408.8 ± 24.9	0.104
OATP1B1	14.9 ± 5.0	844.4 ± 430.5	0.018
OATP2B1	20.1 ± 2.7	284.6 ± 84.3	0.071

a Enalaprilat transport was examined in HEK293 cells transfected with human organic anion transporters (OATs) and human organic anion transport peptides (OATPs). Transporter specific uptake was calculated by subtracting mock cell concentrations from the total at each time point. Data were the average of three separate incubations and presented as mean ± sd.

b Enalaprilat concentrations were 0, 1, 3, 10, 30, 100, and 500 μM; the incubation time was 5 min.

c CL_int_ (intrinsic uptake clearance) was calculated as the ratio of V_max_ over K_m_.

### Inhibition of OAT3 by Selected Drugs

**Figure 2:**
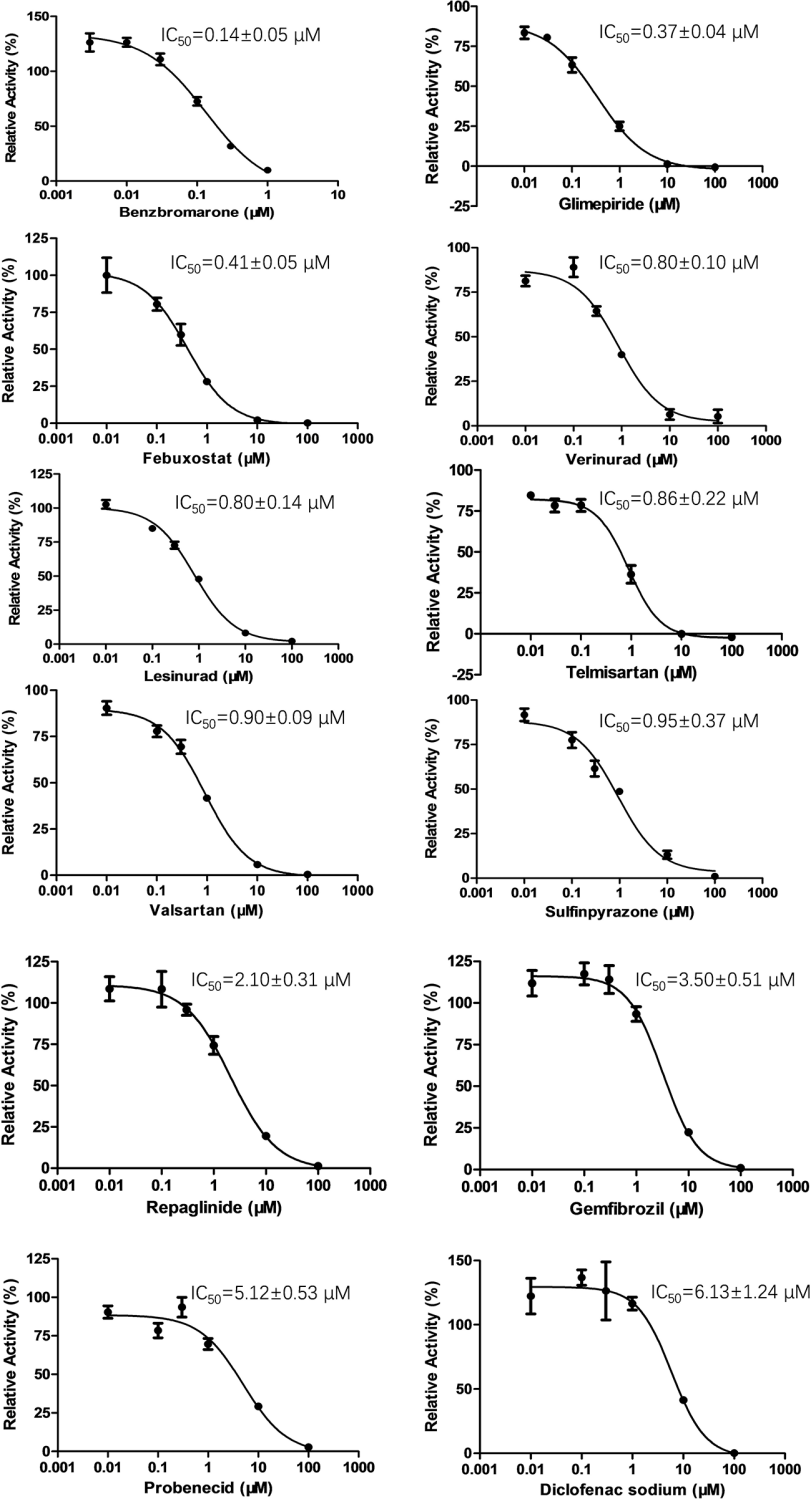
Inhibition of enalaprilat uptake by selected drugs in HEK293 cells transfected with OAT3. Enalaprilat concentration was 30 µM. The concentrations of drugs were as follows: benzbromarone (0.003, 0.01, 0.03, 0.1, 0.3, 1 μM); glimepiride and telmisartan (0.01, 0.03, 0.1, 1, 10, 100 μM); diclofenac sodium, febuxostat, gemfibrozil, lesinurad, probenecid, repaglinide, sulfinpyrazone, valsartan, and verinurad (0.01, 0.1, 0.3, 1, 10, 100 μM). Data were averaged values of three separate incubations and presented as mean ± sd.

To determine the effect of several commonly used drugs on OAT3-mediated enalaprilat uptake, the inhibition of OAT3 transport activity was investigated. As shown in **Figure 2**, benzbromarone (an anti-hyperuricemia agent, IC_50_ = 0.14 µM) was the most potent inhibitor against OAT3-mediated uptake of enalaprilat, while diclofenac (an anti-inflammation agent, IC_50_ = 6.13 µM) was the weakest inhibitor among the tested drugs.

It was interesting to note that OAT3 (SLC22A8) and urate transporter 1 (URAT1) (SLC22A12) belong to the SLC22A subfamily of organic anion transporters (Roth et al., 2012). Therefore, URAT1 inhibitors used for the treatment of hyperuricemia might inhibit OAT3 activity due to protein structural homology. Indeed, commercially available URAT1 inhibitors (benzbromarone, lesinurad, and verinurad) potently inhibit OAT3-mediated enalaprilat uptake, with IC_50_ values <1 µM, except probenecid (IC_50_ = 5.12 µM). The above results suggested a lack of selectivity for URAT1 inhibitors against SLC22A subfamily member OAT3.

### Inhibition of OAT3 by Selected Flavonoids

Effects of flavonoids representing different structural features on OAT3-mediated enalaprilat uptake were examined. As summarized in **Table 2**, inhibitory potency of these selected flavonoids varied widely, with IC_50_ values ranging from 0.03 to 22.6 µM. The structural diversity of these flavonoids and the wide range of inhibitory potency prompted a further computational analysis in order to identify structural features for OAT3 inhibition.

**Table 2:** Inhibition of OAT3-mediated enalaprilat uptake by selected flavonoids.

Name	Structure	IC_50_ (µM)
Galangin	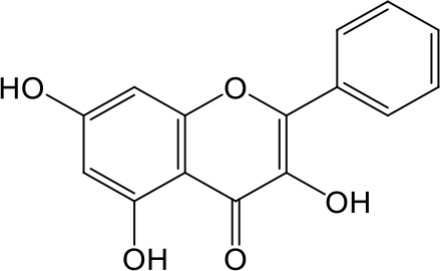	0.030 ± 0.026
Chrysin	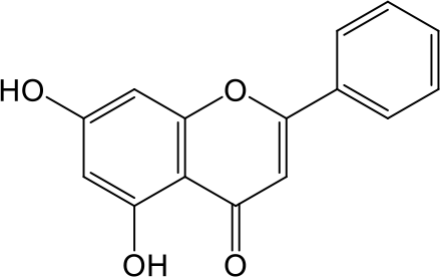	0.044 ± 0.013
Kaempferol	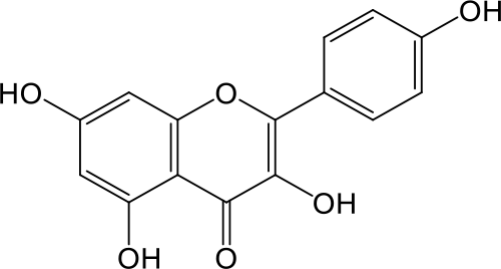	0.088 ± 0.014
Oroxylin A	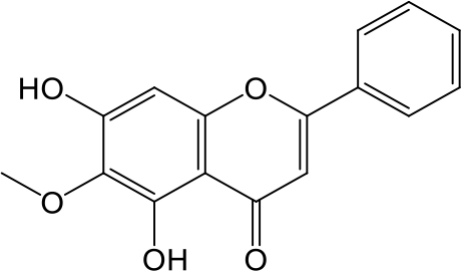	0.22 ± 0.12
Wogonin	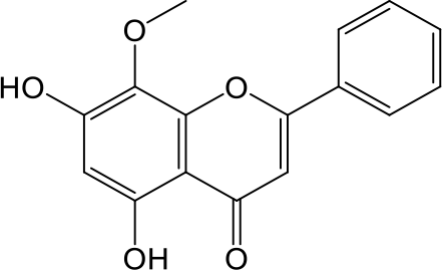	0.24 ± 0.04
Apigenin	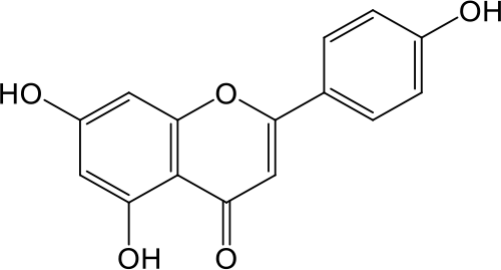	0.33 ± 0.09
Luteolin	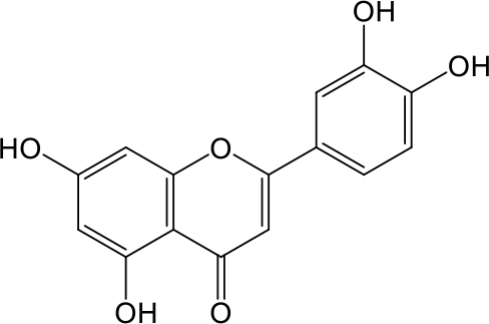	0.66 ± 0.11
Gossypetin	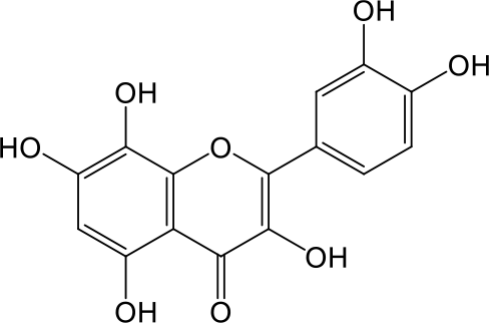	0.71 ± 0.08
Quercetin	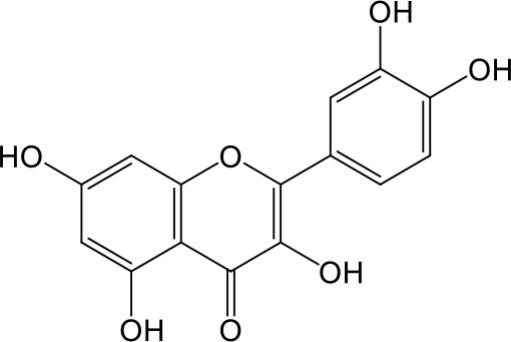	0.75 ± 0.37
Mulberrin	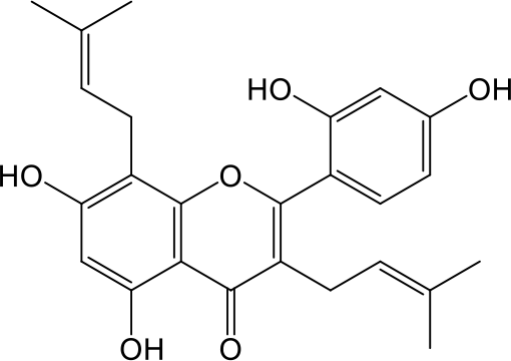	1.22 ± 0.18
Eriodictyol	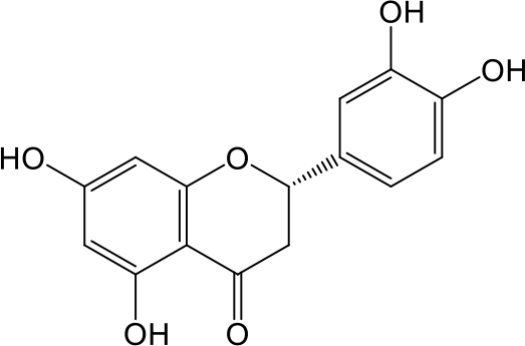	1.48 ± 0.60
Fisetin	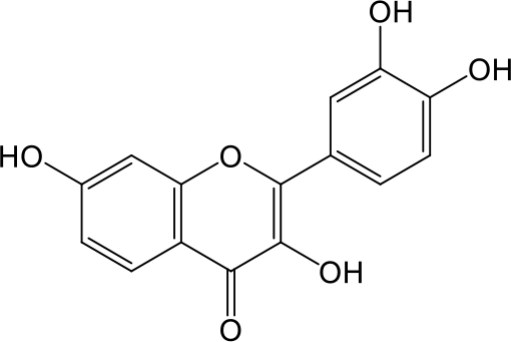	3.82 ± 1.60
Daidzein	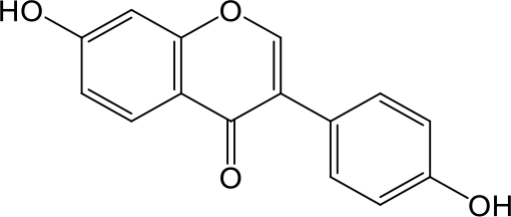	5.80 ± 3.96
Taxifolin	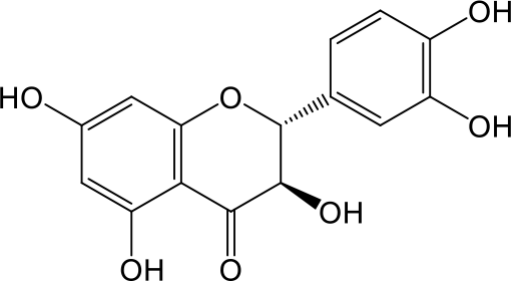	7.02 ± 3.37
Apigetrin	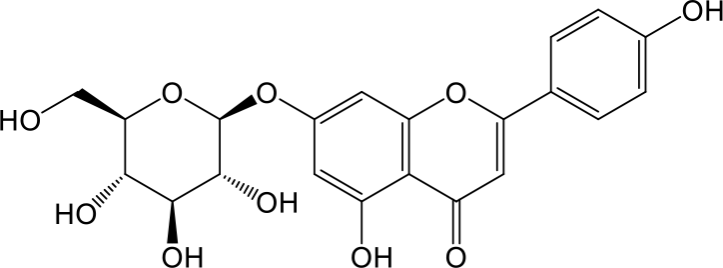	7.80 ± 4.03
Isoquercetin	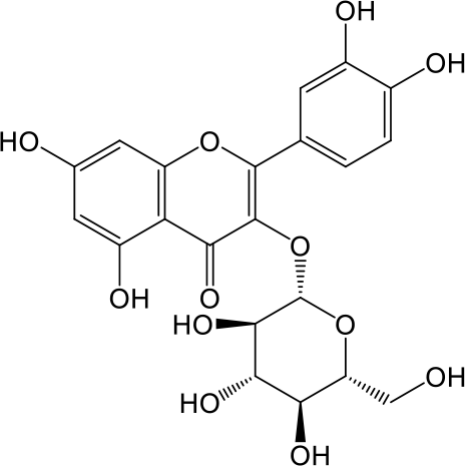	11.71 ± 2.06
Wogonoside	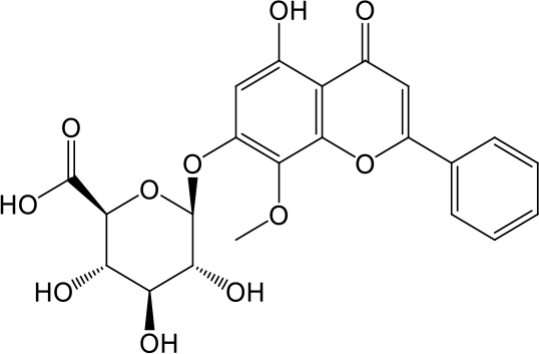	11.73 ± 6.29
Myricetin	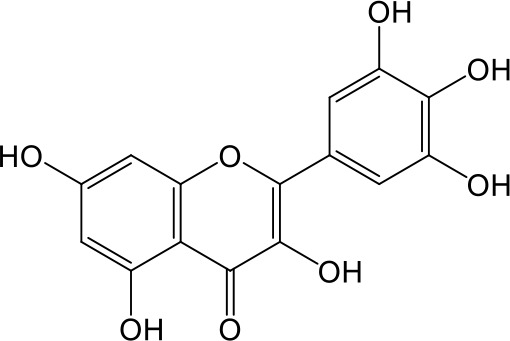	22.58 ± 6.86

Incubations were carried out in HEK293 cells transfected with human OAT3. Enalaprilat concentration was 10 µM and inhibitor concentrations ranged from 0.003 to 100 µM. Flavonoids were listed based on inhibition potency from strong (low IC_50_) to weak (high IC_50_). Data were averaged values of three separate incubations and presented as mean ± sd.

### Pharmacophore Modeling for Selected Flavonoids and Drugs

**Figure 3:**
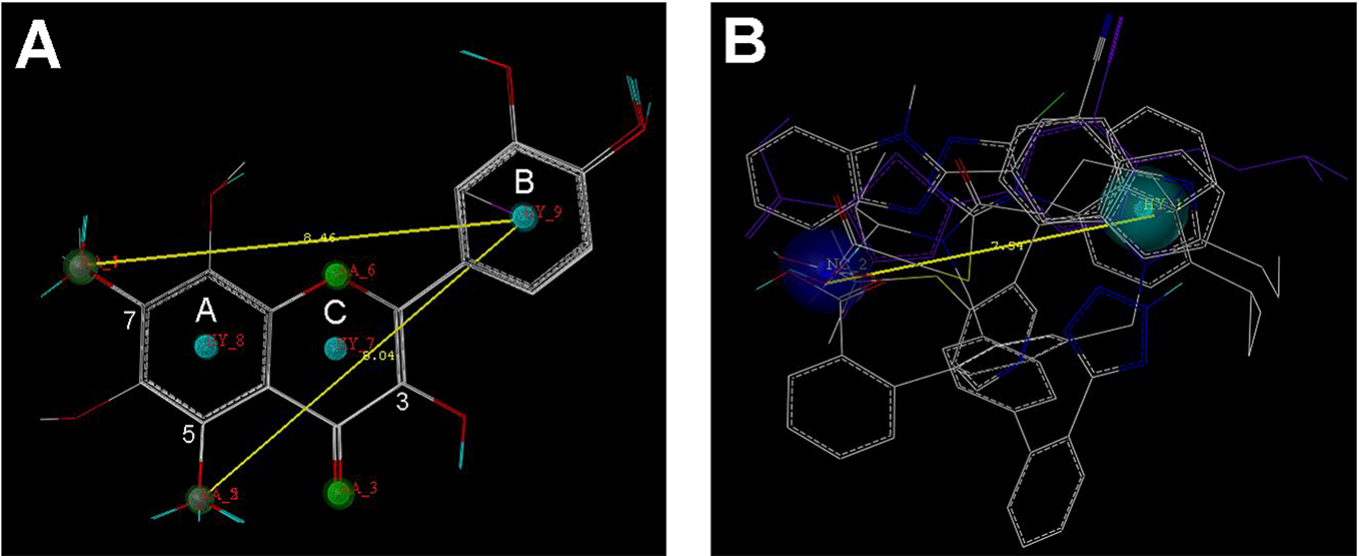
The pharmacophore models for the selected 9 flavonoids and 5 organic acid drugs. **(A)** The GALAHAD model for flavonoids included four hydrogen-bond acceptor atoms (green), two hydrogen-bond donor atoms (magenta), and three hydrophobic centers (cyan); **(B)** The GALAHAD model for selected drugs included one negative center (blue) and one hydrophobic center (cyan). The sphere sizes indicated query tolerances.

Nine flavonoids and five organic acid drugs with strong inhibitory activity against OAT3 (IC_50_ < 1 µM) were selected to generate pharmacophore models for flavonoids and drugs, respectively. Twenty pharmacophore models were generated by GALAHAD for each set of inhibitors. The models with low energy, high specificity, and high steric effects were selected. As shown in **Figure 3A**, the pharmacophore model for flavonoids included four hydrogen-bond acceptor atoms (green), two hydrogen-bond donor atoms (magenta), and three hydrophobic centers (cyan). Therefore, the 5-OH and 7-OH groups on ring A of flavonoids could be both hydrogen-bond acceptor and hydrogen-bond donor groups. The pharmacophore model for selected drugs included one negative center (blue) and one hydrophobic center (cyan) (**Figure 3B**). According to these two pharmacophore models, it appeared that a good OAT3 inhibitor should have a polar center (a negatively charged or hydrogen-bond forming group) and a hydrophobic center with a distance of ~8 Å. For flavonoids, the polar center was most likely a hydroxyl group on ring A and the hydrophobic center was the aromatic ring B (**Figure 3A**).

### 3D-QSAR Analysis on Selected Flavonoids

To further characterize the structure-activity relationship of flavonoids as OAT3 inhibitors, 3D-QSAR studies were carried out with CoMFA and CoMSIA analyses. A total of 18 flavonoids were used with inhibitory potency evenly distributed and covered a range of three orders of magnitude (**Table 2**). The distribution and range of these activity data indicated that they were suitable for CoMFA and CoMSIA analyses.

**Figure 4:**
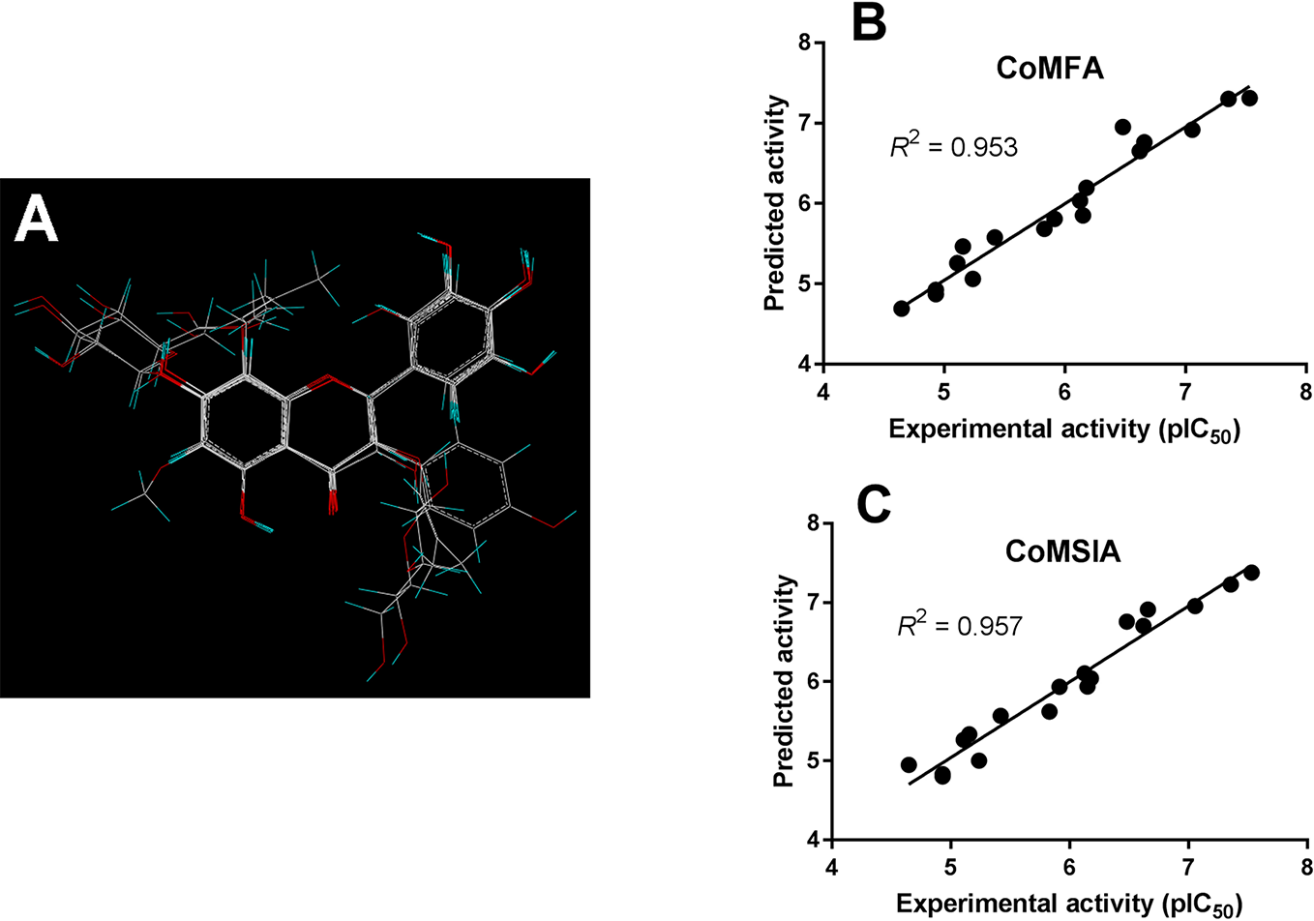
CoMFA and CoMSIA modeling on flavonoids. **(A)** Structural alignment of the 18 flavonoids and the correlation validation between the predicted activities and experimental activities (pIC_50_) for **(B)** CoMFA and **(C)** CoMSIA models.

As shown in **Figure 4A**, the structural alignment of the 18 flavonoids was first obtained based on their three-ring scaffold. CoMFA and CoMSIA models were then developed with PLS analysis and their statistics were listed in **Table 3**. The q^2^ values for CoMFA and CoMSIA were 0.601 and 0.618, respectively. The optimal number of components for both CoMFA and CoMSIA was four. The non-cross validation PLS analysis with the optimal components of four revealed conventional (non-cross validated) r^2^ of 0.953 and 0.957 for CoMFA and CoMSIA, respectively. The standard errors of estimates for CoMFA and CoMSIA were 0.217 and 0.207, respectively. In the CoMFA model, steric fields contributed 34.5% to the model’s information, while electrostatic fields contributed to the other 65.5%. In the CoMSIA model, the contributions of steric, electrostatic, hydrophobic, hydrogen-bond donor, and hydrogen-bond acceptor fields were 8.6, 27.1, 15.6, 28.3, and 20.3%, respectively. The correlation coefficients between the predicted activities and the experimental values (pIC_50_) for CoMFA and CoMSIA models were 0.953 and 0.957, respectively (**Figures 4B, C**).

**Table 3:** Statistical parameters of CoMFA and CoMSIA models for the 18 flavonoids as OAT3 inhibitors.

	CoMFA	CoMSIA
PLS statistics		
q^2^	0.601	0.618
r^2^	0.953	0.957
Standard error of estimate	0.217	0.207
Optimal number of components	4	4
Field contributions (%)		
Steric	34.5	8.6
Electrostatic	65.5	27.1
Hydrophobic		15.6
Hydrogen-bond donor		28.3
Hydrogen-bond acceptor		20.3

**Figure 5:**
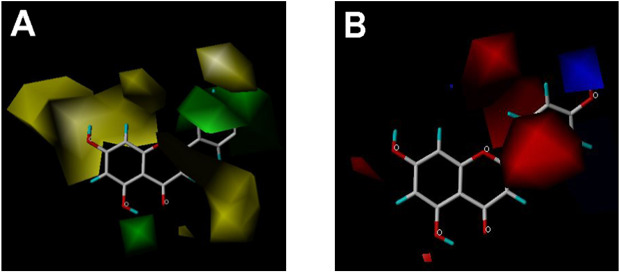
CoMFA contour maps in combination with apigenin. **(A)** Steric field: green, steric bulk desirable regions; yellow, steric bulk undesirable regions. **(B)** Electrostatic field: blue, positive charge desirable regions; red, negative charge desirable regions.

**Figure 6:**
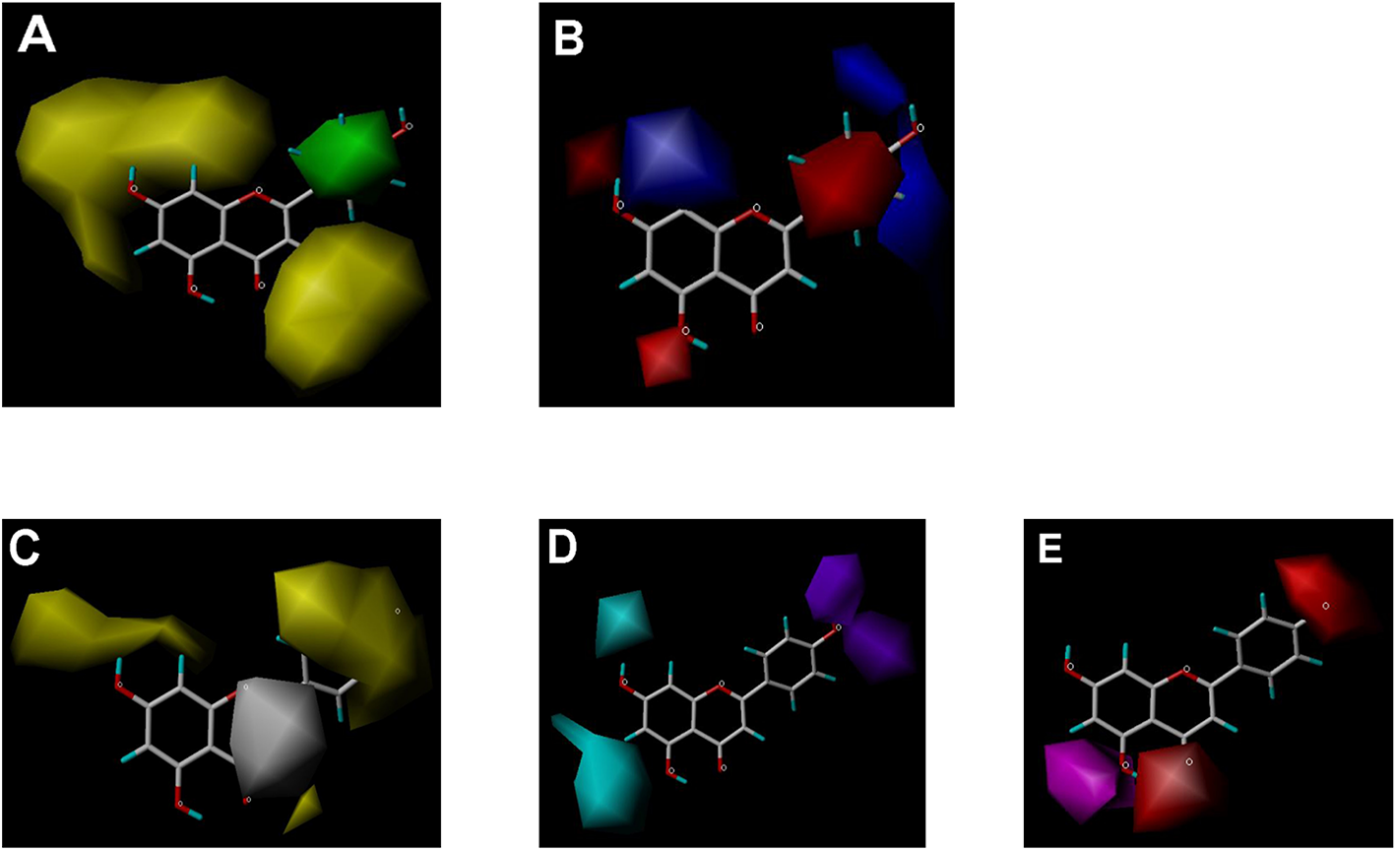
CoMSIA contour maps in combination with apigenin. **(A)** Steric field: green, steric bulk desirable regions; yellow, steric bulk undesirable regions. **(B)** Electrostatic field: blue, positive charge desirable regions; red, negative charge desirable regions. **(C)** Hydrophobic field: yellow, hydrophobicity desirable regions; white, hydrophobicity undesirable regions. **(D)** Hydrogen-bond donor field: cyan, donor desirable regions; purple, donor undesirable regions. **(E)** Hydrogen-bond acceptor field: magenta, acceptor desirable regions; red, acceptor undesirable regions.

CoMFA and CoMSIA steric contour maps showed that regions near C-3 of ring C and C-7 of ring A of flavonoids were steric undesirable (yellow contours in **Figures 5A** and **6A**), indicating that bulky substituents in these regions were unfavorable for flavonoid’s interaction with OAT3. This could explain the inhibition potency difference between quercetin (IC_50_ = 0.75 µM) and isoquercetin (IC_50_ = 11.71 µM), a 3-O-glucoside of quercetin. Similarly, apigenin (IC_50_ = 0.33 µM) and wogonin (IC_50_ = 0.24 µM) showed stronger inhibitory activities than apigetrin (apigenin-7-O-glucoside) (IC_50_ = 7.8 µM) and wogonoside (wogonin-7-O-glucuronide) (IC_50_ = 11.73 µM), respectively.

CoMSIA hydrophobic contour map showed that there was a large hydrophobicity desirable region around the ring B of flavonoids (yellow contour in **Figure 6C**), suggesting that hydrophilic substituents such as hydroxyl group on ring B were unfavorable. This might be the reason why chrysin, apigenin, and luteolin had decreasing inhibitory activities with IC_50_ values of 0.044, 0.33, and 0.66 µM, respectively. The numbers of hydroxyl groups on ring B were 0, 1, and 2 for chrysin, apigenin, and luteolin, respectively, while the remaining parts of their structures were the same. Similarly, galangin, kaempferol, quercetin, and myricetin showed decreasing activities with IC_50_ values of 0.03, 0.088, 0.75, and 22.58 µM, respectively. The only structural difference between those was the numbers of hydroxyl groups on ring B (0, 1, 2, and 3 for galangin, kaempferol, quercetin, and myricetin, respectively). The above results were consistent with the pharmacophore analysis, showing that the ring B of flavonoids was a hydrophobic center for OAT3 inhibitors.

Hydrogen-bond donor and acceptor contour maps also showed that hydrogen-bond donor and acceptor groups on ring B were unfavorable for flavonoid’s activity (purple contour in **Figure 6D** and red contour in **Figure 6E**). Meanwhile, a hydrogen-bond donor desirable region was present in the area of 7-OH of ring A (cyan contour in **Figure 6D**) and a hydrogen-bond acceptor undesirable contour was observed near 5-OH of ring A (red contour in **Figure 6E**), indicating that the polar center of flavonoids identified by pharmacophore modeling (**Figure 3A**) was more likely to be the 7-OH instead of 5-OH on ring A. In addition, a hydrogen-bond donor desirable region was observed near C-5 and C-6 of ring A (**Figure 6D**), which might explain why quercetin (IC_50_ = 0.75 μM) had higher inhibitory potency than fisetin (IC_50_ = 3.82 μM) as quercetin has an additional 5-OH on ring A when compared to fisetin.

## Discussion

Enalaprilat is the active metabolite of prodrug enalapril, a widely used ACE inhibitor for the treatment of general hypertension and heart failure. After oral administration, enalapril is converted to enalaprilat and both enalapril (18% of the dose) and enalaprilat (43% of the dose) are excreted into the urine, suggesting the importance of renal clearance in the disposition of the drug and its active metabolite (Ulm et al., 1982; MacFadyen et al., 1993). Enalapril is used orally with a starting dose of 5 mg daily, whereas enalaprilat (1.25 mg) can only be administered intravenously under the supervision of healthcare professionals for acute hypertension. Although enalapril is well tolerated and safe under normal circumstances, the drug has been shown to have side effects in certain populations. For example, a prolonged use of enalapril during pregnancy may lead to fetal and neonatal injury (Tabacova and Kimmel, 2001). In addition, concomitant use of aliskiren and enalapril maleate should be avoided in patients with renal impairment due to acute kidney toxicity (Yamauchi et al., 2012). Unlike small molecule drugs developed recently, the involvement of transporters in the disposition of older drugs such as enalapril and enalaprilat needs to be evaluated in order to avoid side effects due to herb-drug and drug-drug interactions.

In the present study, transmembrane transport of enalaprilat was investigated in cell lines stably transfected with human uptake transporters and kinetic parameters K_m_ (substrate affinity) and V_max_ (transport capacity) were obtained (**Table 1**). In order to rank the importance of various uptake transporters, the ratio of V_max_/K_m_ (uptake clearance) was used and the results indicated that OAT3 was the predominant uptake transporter for enalaprilat, followed by OAT1 (3 times less), OATP2B1 (4 times less), and OATP1B1 (17 times less). Literature reports suggest that K_m_ values of OAT3 substrates vary widely, ranging from single digit µM to mM. Many OAT3 substrates with high K_m_ values have demonstrated *in vivo* interactions due to inhibition. Those substrates include but not limited to steviol acyl glucuronide with K_m_ of 368.1 µM (Zhou et al., 2020), mesna with K_m_ of 390 µM (Cutler et al., 2012), and isoniazid with K_m_ of 233.7 µM (Parvez et al., 2018). Judging from quantitative proteomic data, both OAT3 and OAT1 are highly and equally expressed in human kidney, whereas OATPs are below detection limits (Prasad et al., 2016; Uchida et al., 2016). Since OAT3 plays an important role in renal excretion of many drugs and endogenous substances (Nigam, 2018), the present results in conjunction with literature reports suggest that OAT3-mediated enalaprilat uptake may be an important mechanism in its renal clearance and the inhibition of OAT3 activity may alter pharmacokinetic and efficacy of enalaprilat. It should be recognized, however, that other transporters such as multidrug and toxin extrusion proteins may act concertedly with OATs in the renal clearance of enalaprilat (Lai et al., 2010).

Data obtained in the present study indicated that OAT3-mediated enalaprilat uptake could be inhibited by a number of drugs and flavonoids with varying degrees of inhibitory potency (**Figure 2** and **Table 2**). For example, commonly used drugs for the control of hypertension (telmisartan and valsartan), hyperglycemia (glimepiride), and hyperuricemia (febuxostat, benzbromarone, etc.) displayed potent inhibition of OAT3-mediated uptake of enalaprilat, suggesting an interaction potential due to OAT3 inhibition. In fact, OAT3 as a molecular target for drug-drug interactions have been reported. For instance, it was shown that inhibition of OAT3-mediated pemetrexed uptake by lansoprazole would exacerbate pemetrexed-mediated hematologic toxicity (Ikemura et al., 2016). Similarly, inhibition of OAT3-mediated methotrexate uptake by proton pump inhibitors could result in a pharmacokinetic interaction that increases the plasma methotrexate levels (Narumi et al., 2017). It is thus possible that the inhibition of OAT3-mediated enalaprilat uptake by some commonly used drugs may lead to clinically relevant drug-drug interactions.

Among the solute carrier (SLC) superfamily of uptake transporters, OAT3 (SLC22A8) and URAT1 (SLC22A12) belong to the SLC22A subfamily (Lozano et al., 2018; Schaller and Lauschke, 2019). Because of high protein sequence homology, it can be postulated that URAT1 inhibitors developed as antihyperuricemia drugs are likely to inhibit OAT3 transport activity. Indeed, results from the present study demonstrated that all URAT1 inhibitors tested displayed potent inhibition against OAT3-mediated enalaprilat uptake. The lack of selectivity of URAT1 inhibitors against SLC22A subfamily members may be a clinically important consideration for renal homeostasis, as cross inhibition of these closely related transporters may exacerbate herb/drug-drug interactions. As the transporters in SLC22A subfamily play critical roles in health and diseases, the inhibition of OAT3-mediated enalaprilat transport by URAT1 inhibitors should be investigated in the clinical setting (Lai et al., 2018; Nigam, 2018). In addition, some URAT1 inhibitors such as probenecid, benzbromarone, lesinurad, and verinurad could also inhibit OAT1 and OAT4 (Ahn et al., 2016; Miner et al., 2016; Tan et al., 2017). However, the observed inhibitory potencies on OAT1 and OAT4 were quite different from that on URAT1. For instance, verinurad inhibited OAT1 and OAT4 with approximately 200-fold lower affinity as compared to URAT1 (Tan et al., 2017).

Flavonoids investigated in the present study displayed a wide range of inhibition potency against OAT3-mediated enalaprilat transport. Pharmacophore and 3D-QSAR analyses revealed the presence of a polar center and a hydrophobic center for OAT3-inhibitor binding. For the polar center, the binding between flavonoids and OAT3 could be readily characterized by the position and number of hydrogen bond donors/acceptors in flavonoids (**Figures 3**–**6**). Further correlation analysis demonstrated that the predicted inhibitory potencies by 3D-QSAR models matched well with experimental IC_50_ values (**Figure 4**). The above findings suggest that the current pharmacophore and 3D-QSAR models could be useful to explore structure-activity relationship of flavonoids and other structurally related compounds as OAT3 inhibitors. The models developed in the present study might be predictive for other OAT3 substrates and inhibitors if those compounds share key structural attributes with enalaprilat. Because OAT3 is a multi-specific transporter, caution should be taken when a structurally unrelated substrate is used as a transport probe since it could have a different binding site/mode with OAT3 as compared to enalaprilat. Indeed, a previous report using machine learning described that a pharmacophore model developed from a group of OAT3 ligands even possessed cationic characteristics (Liu et al., 2016), indicating that large differences between different OAT3 ligands may exist.

In conclusion, the present study identified OAT3 as an important uptake transporter for enalaprilat, the active metabolite of enalapril maleate. A number of flavonoid and drug molecules displayed potent inhibition against OAT3-mediated enalaprilat uptake. As such, inhibition of OAT3-mediated renal excretion should be carefully evaluated for not only enalaprilat, but also other OAT3 substrates as it represents an important underlying mechanism for drug/herb-drug interactions. Computational analysis revealed the presence of a polar center and a hydrophobic region with which the inhibition potency could be predicted for flavonoids. The current findings help raise the awareness of the potential of OAT3-mediated herb/drug-drug interactions and caution should be exercised when various remedies of OAT3 substrates and inhibitors are used concomitantly.

## Data Availability Statement

All datasets generated for this study are included in the article/**Supplementary Material**.

## Author Contributions

Conceptualization: CG and HZ. Data curation: YN, ZD, SL, and HW. Formal analysis: YN and ZD. Investigation: YN, ZD, DZ, SL, and HW. Methodology: YN, ZD, SL, and HW. Resources: DZ. Software: CG. Supervision: HZ. Visualization: ZD and CG. Writing—original draft: YN, CG, and H.Z. Writing—review and editing: YN, CG, and HZ.

## Funding

The current work was supported in part by a research grant (#81473278) from National Natural Science Foundation of China.

## Conflict of Interest

The authors declare that the research was conducted in the absence of any commercial or financial relationships that could be construed as a potential conflict of interest.

## Abbreviations

OAT3, human organic anion transporter 3; OCT2, human organic cation transporter 2; OATP, human organic anion transporting polypeptide; URAT1, human urate transporter 1; HEK293, human embryonic kidney cells; 3D-QSAR, three-dimensional quantitative structure-activity relationship; CoMFA, comparative molecular field analysis; CoMSIA, comparative molecular similarity indices analysis.
