# Supplementary material for: Identification of Structural Features for the Inhibition of OAT3-Mediated Uptake of Enalaprilat by Selected Drugs and Flavonoids
Source: Front Pharmacol. 2020 May 28;11:802. doi: 10.3389/fphar.2020.00802 (PMC7271668; doi:10.3389/fphar.2020.00802)
Supplement: Supplementary file 1 [file Image_1.pdf]

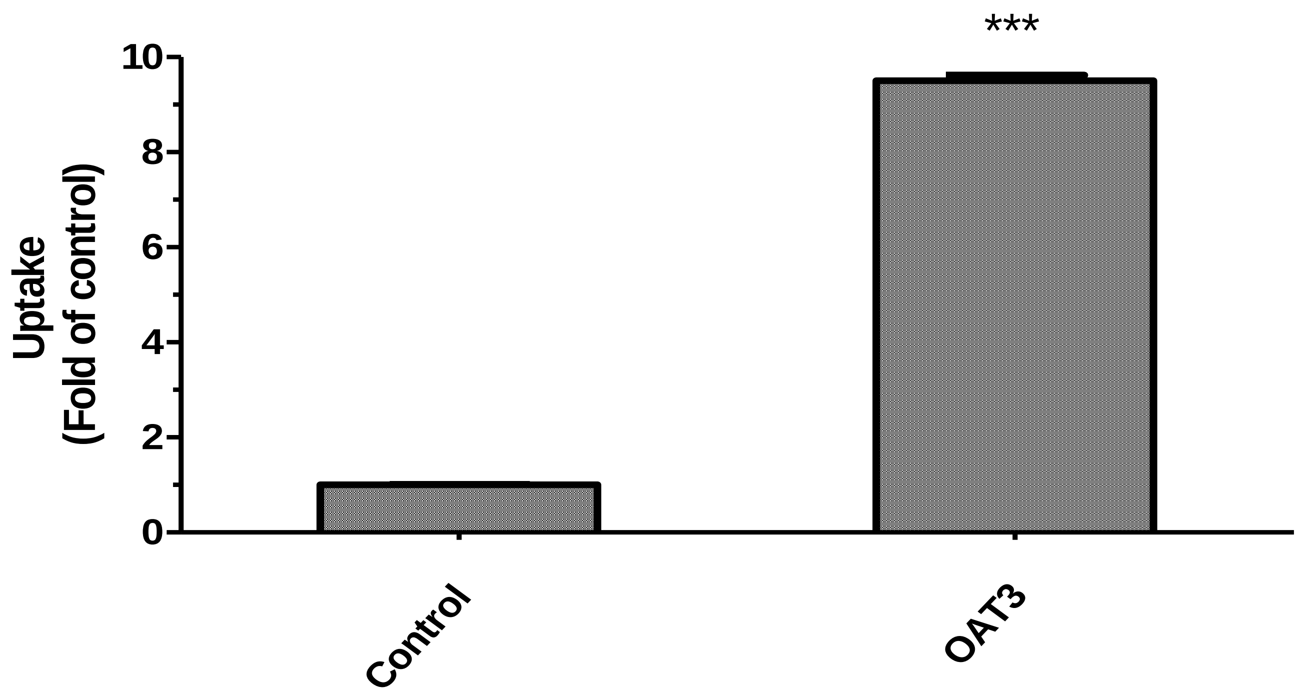

Supplementary Figure 1. Uptake of enalapril in OAT3-transfected HEK293 cells. Enalapril concentration was 10 μM and incubation time was 5 min. Data were presented as mean ± sd. ***P<0.001.
